# Research Review on Teacher Emotion in Asia Between 1988 and 2017: Research Topics, Research Types, and Research Methods

**DOI:** 10.3389/fpsyg.2019.01628

**Published:** 2019-08-21

**Authors:** Junjun Chen

**Affiliations:** Department of Education Policy and Leadership, The Education University of Hong Kong, Tai Po, Hong Kong

**Keywords:** teacher emotion, Asia, research foci, research type, research method, review

## Abstract

**Background:** Studies on teacher emotion have steadily become more prominent. However, it has been observed that scholarship on teacher emotion has been dominated by contributions from Western societies. In the absence of a critical mass of empirical research generated from within the region such as Asia, lack of knowledge and capacity to inform policy and practice in teacher education and evaluation mechanism.

**Methods:** The current study sought to mirror the patterns of knowledge production in teacher emotion in terms of research topics, types, and methods and how they have evolved over time in Asia between 1989 and 2018. By using a descriptive quantitative analysis approach, we review a corpus of 154 articles on teacher emotion published in peer-review journals in Asia. It began with a systematic search in seven widely used electronic databases for abstracts of potentially relevant studies.

**Results:** As observed, the trend represents a sea change in the volume of publications in Asia although the overall volume of research is still relatively low. Results also identify that although quantitative methods were most commonly used, the findings reveal a more balanced distribution since 1993. Furthermore, the use of qualitative and mixed methods of research has undergone a marked increase in the past 10 years. However, as the majority of articles were exploratory-oriented, intervention and experimental studies were largely lacking, which resulted in the ‘so-what' story being missing.

**Conclusions:** A functionalist perspective suggests that knowledge production in teacher emotion research in Asia is either at the late first stage or the emerging second stage. Recommendations have been provided based on the major results and challenges.

## Introduction

In recent years, studies on teacher emotion have steadily become more prominent. However, it has been observed that scholarly works on teacher emotion has been dominated by contributions from Western societies (Uitto et al., 2015). In the absence of a critical mass of empirical research generated from within regions, such as Asia, scholars, educators, and policymakers have only a limited understanding of how teacher emotion is developed and practiced outside of the “Western academic mainstream” and, therefore, lack the knowledge, and capacity to inform policy, and practice for teacher education, and evaluation mechanisms. However, globalization in educational research, including teacher emotion, tends to ignore the social-cultural context which can “act as a mediator or filter to the spread of ideas and practices across the globe resulting in their adoption, adaptation or even rejection” (Dimmock and Walker, 2000, p. 304, cited in Hallinger and Bryant, 2013). The often-dysfunctional results of policy and practice borrowed from the West in Eastern societies have already been proven (e.g., Tan and Chua, 2015; Qian et al., 2017). Research results from these studies show that Western medicine cannot cure Easter diseases.

Practitioners in Asian contexts have also rebounded the concern for emotional knowledge and support (Chen, 2018). This need was emphasized by scholars saying that teachers are ill-prepared and insufficiently supported by initial teacher education and professional training programs on how to handle emotional difficulties in their personal and professional lives (Darling-Hammond, 2001; Gallant, 2013; Hoy, 2013) as well as closeness and conflicts with students (Longobardi et al., 2016). Therefore, building a knowledge base on teacher emotion in the Asian region seems timely and greatly needed.

A global knowledge base on teacher emotion would be capable of providing a more granular understanding of how teachers meet the emotional challenges of dealing with their emotions and how these emotions impact classrooms across different organizational and socio-cultural contexts, and how research could support different stakeholders. As noted, persistence in examining a research issue through a combination of sustained theoretical and empirical programmatic investigations is required in order to produce knowledge accumulation and breakthroughs in understanding (Heck and Hallinger, 2005).

The trend of applying more powerful and diverse conceptual and methodological tools to the investigation of teacher emotion has evolved over time (Murphy et al., 2007; Hallinger and Chen, 2015). Conceptual tools include the explicit elaboration and application of more diverse theoretical models to scholarly work on teacher emotion. Methodological advancements have centered on the use of more systematic approaches in carrying out research for the next. This is observable in the means of reviewing the various kinds of studies in a given field (Hallinger, 2013).

### Rational of Investigating Teacher Emotion in the Asian Context

Teacher emotion is currently considered to be a vital research focus due to the following reasons. First, as mentioned previously, teacher emotions are affected by different contexts—cultural, organizational political, and social (Fried et al., 2015). Given the context-dependent feature of teacher emotion, people in Asia have a different understanding of emotion compared to people in the West (Sundararajan, 2015). Since research on emotions is still dominated by Western studies, unique emotional differences existing in Eastern and Western contexts have pointed to the need for more research studies being conducted in this area, to comprehend how varying contexts and cultures form interactions between different teacher emotions and contexts.

Second, teacher emotion plays a crucial role in teacher well-being (Fried et al., 2015). Teachers nowadays experience more negative emotions than positive ones (Thompson, 2014; Hassard et al., 2016) and teachers in Asia have been diagnosed and suffer from the same problem of negative emotions in recent years (Chen, 2019). Negative emotions and an inadequate capacity of emotion regulation continues to rank as one of the major reasons for teacher burnout and for teachers leaving their profession (Akin et al., 2013).

Third, teacher education and evaluation in most countries in Asia have been driven by increasing academic accountability which is renowned for high-stakes examinations which seem to downplay emotions and solely prioritize achievement (Schutz et al., 2010). Furthermore, other cultural norms and customs (e.g., promotion of high achievers with academic success as public role models and extremely high expectations from parents on their children) might also contribute to this negligence (Li, 2009). Recently, there have been debates on policy discussing the notion of connecting teacher pay with measurements of “merit” in which teachers are evaluated and promoted (Fried et al., 2015). Such a focus has had an impact on the emotional climate of classrooms in a number of ways due to the nature of teacher/student interactions and behaviors that are being influenced (Schroeder, 2006), as well as the performance of students (Srinivasan, 2015). Research suggests an increased emphasis on high-stakes testing and accountability is changing the nature of classroom transactions (Schutz et al., 2010), which is associated with increased teacher attrition (Behrent, 2009), and teacher stress (Valli and Buese, 2007), pressure and anxiety (Thompson, 2014). This has not helped the existing fragile emotional state of teachers (Thompson, 2014).

Fourth, research has identified a significant impact of teacher emotion on the learning and teaching process. On the one hand, researchers have identified that teacher emotions are linked to various classroom aspects such as students' emotions (van Uden et al., 2014), student-teacher relations (Yan et al., 2011), and academic outcomes (Srinivasan, 2015). For example, it has been identified that teacher support affects closeness and conflicts with students (Marengoa et al., 2018) as well as their adjustment in the classroom in typical and atypical development (Longobardi et al., 2016; Prino et al., 2016). On the other hand, others have identified that teachers' cognitive process and cognitive effectiveness is influenced by their emotions, such as their perception, attention, memory, and problem solving (Golombek and Doran, 2014). Findings have demonstrated that maintaining more positive emotions generates more innovative ideas and strategies (Chen, 2018), whereas holding negative emotions in decreases motivation (Sutton and Wheatley, 2003). In addition, teacher emotions have an impact on their personal and professional lives (Schutz and Zembylas, 2009) and ultimately affects teacher effectiveness (Day, 2011).

Fifth, teachers' emotional capacity is malleable. Teachers' emotional capacity refers to identifying and managing their own emotions and the emotions of others. It consists of regulating one's own emotions and cheering up or calming down other people around them (Akbari et al., 2017). Research found that, with appropriate training and interventions, human beings' emotional capacity can be promoted (Siu et al., 2014). Research has shown that teachers with higher emotional regulation capacity tend to better manage their emotions and have a higher level of job satisfaction and well-being (Yin et al., 2017) and better teaching quality (Heydarnejad et al., 2017). In sum, it seems that teacher emotions are empirically and conceptually significant and worth investigating to unveil the true nature of teacher emotion and the trajectory of its knowledge accumulation.

### Objectives

Since there have been no review studies undertaken in Asia, specifically focusing on the developmental trends of research, the current study sought to mirror the patterns of knowledge production in teacher emotion, in terms of research topics, types and methods particularly in the Asian region between 1988 and 2017, and how they have evolved and interacted over the past 30 years. This approach enabled us to compare patterns of intellectual progress in Asia with a more general historical development of the field. We hope that this leads to strategies that accelerate progress in research capacity development and knowledge production in the future (Hallinger and Bryant, 2013).

### Research Questions

The following research questions were formulated and addressed in this review:

1. What was the topical focus of articles published on teacher emotion from Asia and how has it altered between 1988 and 2017?
2. In what ways were research types distributed (e.g., empirical, theoretical, and review) and how have they altered in research published in Asia between 1988 and 2017?
3. Which research methods did scholars employ when studying teacher emotion and how have they changed in research published in Asia between 1988 and 2017?
4. How were research topics related to research on teacher emotion literature from Asia and in what ways have they changed between 1988 and 2017?
5. How were research topics related to research methods on teacher emotion literature from Asia and in what ways have they changed between 1988 and 2017?

## Methods

### Study Design

A descriptive and quantitative form of a systematic review of published research studies (Gough, 2007; Hallinger, 2011) was used in this study. Over the past 30 years, patterns of change observed in studies conducted in Asian contexts on teacher emotion have been analyzed in research topics, research types, and research methods. Four phases utilized in the review method are discussed in this section, namely; the criteria used for inclusion and exclusion; literature searches and the identification of studies; data screening and extraction; and the analysis of reported data.

### Inclusion Criteria

In order to ensure that the articles retrieved were relevant to the stated aims, prior to the systematic search being conducted, we established inclusion, and exclusion criteria. Therefore, when retrieving articles, the following inclusion criteria were applied. Initially, research topics needed to be of relevance, and focus on emotional experience and/or emotion-related teacher constructs. Second, it was a requirement that articles be published between the years 1988 and 2017. Third, the articles had to be first published on-line in 2017. Fourth, one set of data in empirical articles, at least, needed to be gathered from Asia. Fifth, the first author and/or corresponding author's affiliation in theoretical and/or review articles needed to be from Asia. Sixth, the selected peer-reviewed articles must be published in the English language.

### Search Strategy and Study Identification

We had both historical and pragmatic reasons for selecting this 30-year period. Historically, in the late 1980s and early 1990s, there was a shift from the previously dominant rationalistic approach in which emotional issues were ignored and/or dismissed, considering mood and affect to be interest variations (Ashkanasy et al., 2002). In educational environments, the revitalization of teacher emotion research was lagging behind. Pragmatically, in our initial search for the current review, the earliest article found and published in Asia with teacher emotion research foci emerged in 1990 by Babad. However, we made the conscious decision to select 1988 as the demarcation point for two reasons. First, there may have been some outlier studies from our initial search. Second, having a starting year ensured the study had an integer of 30 years.

The databases searched were ERIC, ProQuest PsycArticles, PsycINFO SAGE, ScienceDirect, Scopus, and Web of Science. The following keywords on teacher emotion were entered, matching the databases' subject headings, or title and abstract keywords. The search terms used in the title, keywords, or abstract were teach^*^ AND Emotion^*^ OR feeling^*^ OR affective^*^ OR mood^*^.

### Data Screen and Extraction

**Figure 1:**
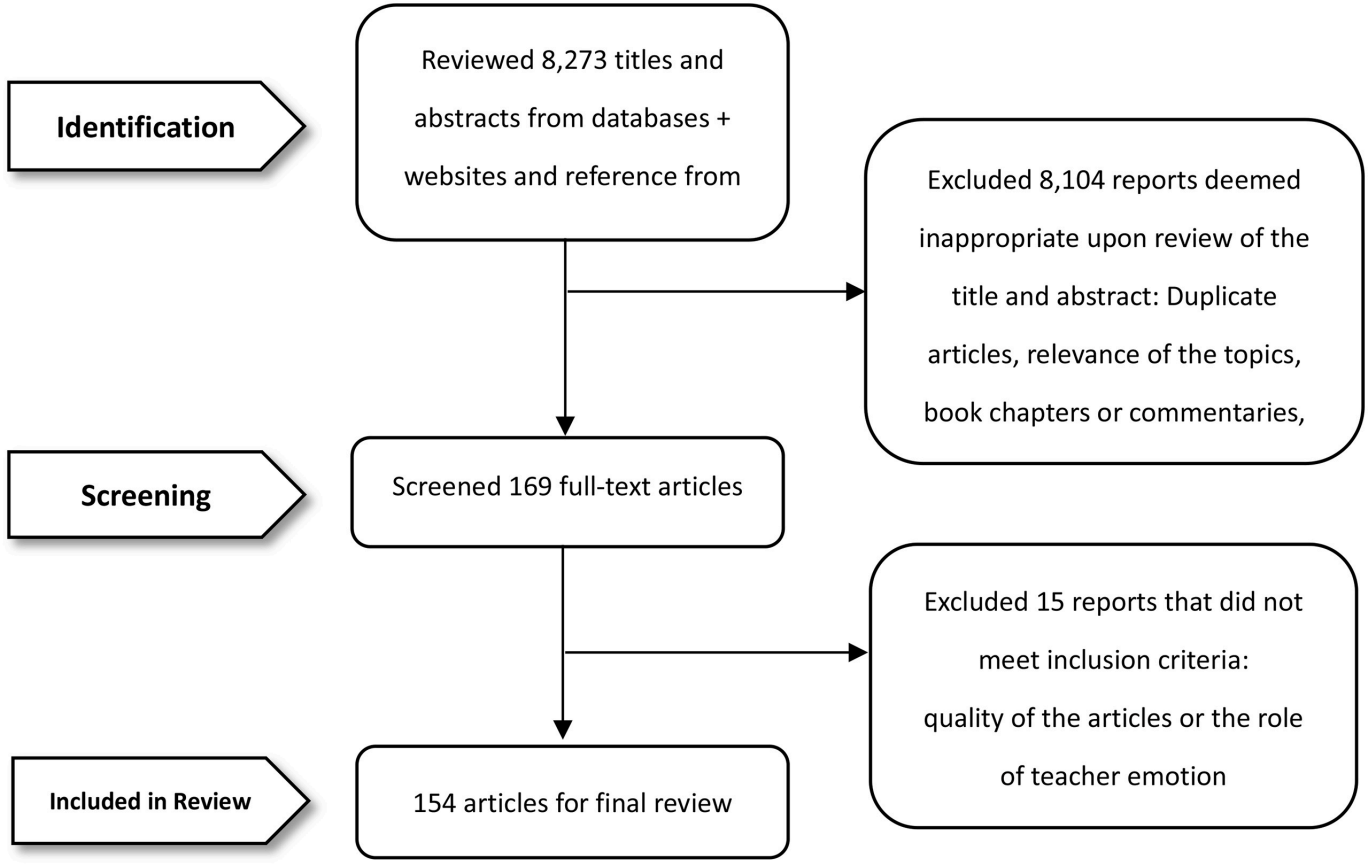
Flow chart of search and screening process.

PRISMA (Preferred Reporting Items for Systematic Reviews and Meta-analyses) guidelines were systematically followed when conducting the systematic reviews of the research studies (Moher et al., 2009). There are three specific steps PRISMA follows and reports when identifying and extracting information. The first step was *identification* by screening the abstracts from seven on-line databases. We particularly thought about factors such as duplication, topic relevance, book chapters or commentaries, and if full articles were written in English. The second step undertaken was *screening*. We considered at this stage whether articles were appropriate, and if questionable, we obtained the full text document and independently screened it. The third and final step was *inclusion*. Close attention was paid to article quality and whether the role of teacher emotion was discussed. Attention was also paid to where data were collected from. A pre-requisite was that articles in the corpus needed at least one set of data collected from Asia. Consequently, a total of 154 articles were yielded for the final analysis (Figure 1).

### Data Analysis

We used the descriptive approach to report patterns that emerged (Sandelowski, 2010), which provided, over a particular period of time, general development patterns and variations on research conducted on teacher emotion (Polanin et al., 2017). First, articles that adhered to the pre-determined inclusion criteria were coded with a self-developed instrument that extracted data using Microsoft Excel. The instrument comprises the following sections: time of publication, location of the collected data, and research topic foci, type and method. These reporting data are presented in the following results section.

## Results

Our search identified 154 articles from and/or about Asia, published between 1988 and 2017. We examined the volume of articles published in Asia during this period and analyzed the distribution of articles by research topics, research types, and research methods, and how research topics were related to the research types and methods.

**Figure 2:**
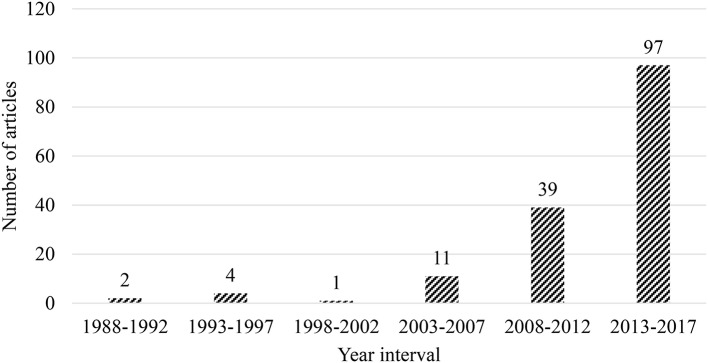
Trend of publication volume by a 5-year interval during 1988–2017 (*n* = 154).

Databases showed that the number of articles continuously increased during the review period (Figure 2). The article located first was published in 1990 (Babad, 1990). Only a couple of articles were available until 2009. Subsequently, an uninterrupted rise was found other than in 2012 and 2016, when two small fluctuations appeared. A peak was reached in 2017, 25 (16.23%). We divided the 30-year duration into six 5-year periods. A continuous increase over the 30-year period was found, and a fluctuation in the third 5-year interval. In the last period, the publications (97, 62.99%) contributed the largest share of the total article corpus. In general, studies conducted in Asia on teacher emotion which have been undertaken over the past 30 years have developed and expanded continuously. There have been significantly more visible contributions made by teacher emotion researchers residing in Asia in the last 10 years. The calculation of data location also yielded an interesting trend. The top five data locations were Turkey (23, 14.94%), Iran (19, 12.34%), Israel (17, 11.04%), Hong Kong (14, 9.10%), and China (13, 8.44%). In addition to the top centers of published research (86, 55.84%) by society, 14 other societies (i.e., Bahrain, Jordan, Singapore, Syria, and Vietnam) (68, 44.16%) also made great contributions to the total research output in Asia.

### Research Topic

Looking initially at the dataset in the current project, the majority of articles focused on exploring the relationship between teachers' emotions and emotional capacity with other relevant constructs and only a small proportion of articles examined the nature of teacher emotion. Out of these relationship studies, there are a set of studies focusing particularly on teachers' emotional capacity rather than teachers' emotions. Meanwhile, learned from the research team's previous review project (Hallinger and Chen, 2015), the relationship studies could be classified into different models. Therefore, we identified three themes regarding teacher emotion, namely, Theme 1–the nature of teacher emotion (32, 20.78%), Theme 2–various models regarding teacher emotion (114, 74.03%), and Theme 3–teachers' emotional capacity (17, 11.04%). First, the nature of teacher emotion consisted of four sub-themes, namely, theories for studying teacher emotion (7, 4.55%), emotion and teacher emotion definitions (2, 1.30%), teacher emotion content (24, 15.58%), and teacher emotion quantitative measures (2, 1.30%). It is interesting to emphasize the definition of emotion from the dataset since it has not yet achieved a consensus over the years (Buric et al., 2017). Although many definitions of emotion have appeared over the years, it seems that scholars from Asia tend to acknowledge to understand emotions from multiple aspects. For example, Borrachero et al. (2014) advocated the one proposed by Kleinginna and Kleinginna (1981) that emotion is a complex set of interactions among subjective and objective factors, mediated by neural systems, which can give rise to affective experiences, generating cognitive processes that can lead to behavior which is often, but not always, adaptive and goal-directed.

Theme 2 referred to various models. We modified a conceptual framework previously developed by Hallinger (2011) on educational leadership and utilized it in the current review. Research studies on teacher emotion were classified into four simple model types in this framework, namely, direct effects, mediated effects, precursor effects, and reciprocal effects (see Table 1).

**Table 1:** Summary of research topic.

**Theme and sub-theme**	**No. of studies**
**Theme 1. Nature of teacher emotion**	32
A. Theories to study teacher emotions	7
B. Definitions of emotion and teacher emotion	2
C. Content of teacher emotion	24
D. Quantitative measures of teacher emotion	2
**Theme 2. Various models regarding teacher emotion**	114
A. Precursors of teacher emotion	39
A1. Malleable precursors	22
A2. Contextual precursors	9
A3. Unmalleable precursors	8
B. Direct effects of teacher emotion	50
B1. Teacher emotion and teachers themselves	35
B2. Teacher emotion and teaching	10
B3. Teacher emotion and student learning	5
C. Mediated effects of teacher emotion	25
C1. Mediated effects of teacher emotion on precursors and outcomes	12
C2. Mediated effects of teacher emotion on precursors and mediating variables	4
C3. Mediated effects of teacher emotion on mediating variables and outcomes	9
D. Reciprocal effects of teacher emotion	0
D1. Reciprocal effects of teacher emotion on precursors and outcomes	0
D2. Reciprocal effects of teacher emotion on precursors and mediating variables	0
D3. Reciprocal effects of teacher emotion on mediating variables and outcomes	0
**Theme 3. Teachers' emotional capacity**	17
A. Content of teachers' emotional capacity	14
B. Measurements of teachers' emotional capacity	3

**Figure 3:**
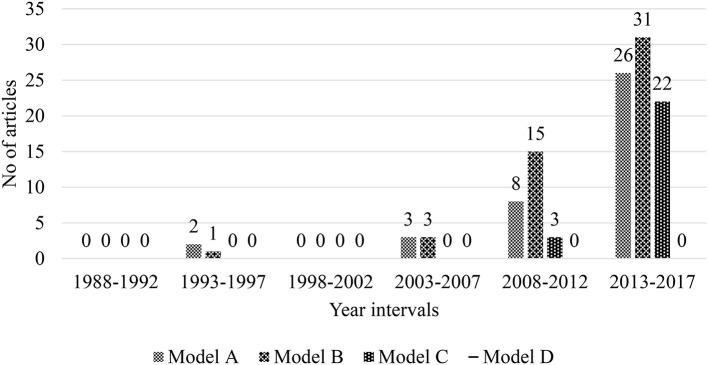
Trend of models of studying teacher emotion by the 5-year interval during 1983–2017 (*n* = 114). Model A stands for the relationship between precursors and teacher emotion; Model B stands for the relationship between teacher emotion and outcomes; Model C stands for the mediated effects of teacher emotion between other constructs; Model D stands for the reciprocal effects of teacher emotion between other constructs.

Published studies that adopted Model A, a precursor-effect model, explored the effects of various precursors on teacher emotion (39, 25.32%). Three kinds of precursors, namely, malleable precursors (22, 19.30%), contextual precursors (9, 7.89%), and unmalleable precursors (8, 7.02%) mainly influenced teacher emotion (Figure 3).

Model B described direct-effect studies of which, an independent variable, teacher emotion, was structured and explored the relationship that existed to one or more dependent variables (50, 32.47%). This part considered mainly teacher emotion and its effect on teachers themselves (35, 22.73%), teaching (10, 6.49%), students, and their learning (5, 3.25%).

Model C consisted of mediated-effect studies (25, 16.23%). The researchers sought to ascertain the routes through which teacher emotion influences distal dependent variables. Such variables were student learning outcomes, teacher efficacy, or teaching method. Any articles located which investigated the mediated effects of teacher emotion on precursors and outcomes were categorized as C1 (12, 7.79%). Those studies that dealt with the mediated effects of teacher emotion on precursors and mediating variables were included in the C2 category (4, 2.60%). Any studies that discussed the mediated effects of teacher emotion on mediating variables and outcomes were categorized as C3 (9, 5.84%).

A reciprocal-effect model of teacher emotion effects was portrayed in Model D. The researchers attempted to recognize the common influence of teacher emotion and its associated variables. It was unsurprising that Model D was not utilized in any of the studies within the teacher emotion literature. Generally speaking, the use of Models A and B were found to dominate across the whole period. Model C also had a central role during the last 5-year interval. Model D was never employed over the given period.

The third theme was about the emotional capacity of teachers with 17 papers (11.04%) consisting of two aspects. First, the content of teacher emotional capacity (14, 9.09%). Second, the measurements of teacher emotional capacity (3, 1.95%).

To sum up, most articles regarding the nature of teacher emotion introduced the content, constructs, and/or elements of teacher emotion, but few of them were concerned with definitions, models, or measures. The utilization of Models A and B (precursor effect models) was continuously strong during the 30-year review period. An increased use of Model C (mediated effect) frameworks was found during the review's final period. Reciprocal-effects studies (Model D) were not identified. There is a possibility that such model types only emerge in research areas which are highly mature. The emotional capacity articles in Theme 3 mainly dealt with the content and measurement of teachers' emotional capacity.

### Research Types

**Figure 4:**
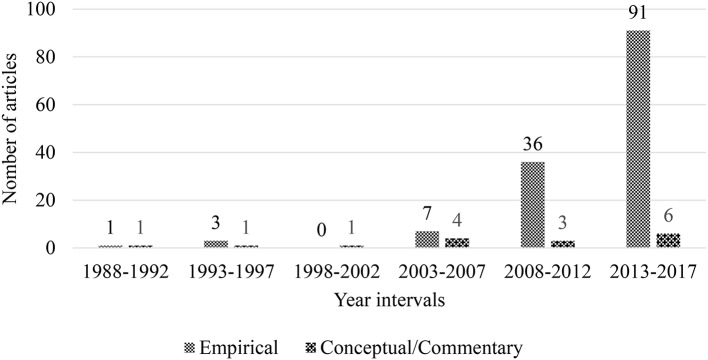
Trend of article type by the 5-year interval during 1983–2017 (*n* = 154).

The research types of articles on teacher emotion were examined next. The three classifications were applied, namely, empirical, review, and conceptual/commentary papers. Empirical studies were data-based research employing quantitative, qualitative, or mixed methods. Review articles may include systematic qualitative reviews, meta-analysis reviews, and science mapping reviews or quantitative descriptions across a body of studies. Conceptual/commentary papers included both theoretical treatises and non-empirical commentaries on issues of policy and practice (Hallinger, 2018). All three types of articles contributed to the development of a mature knowledge base in any field of inquiry. It is apparent that empirical studies are the dominant preference when scholars have undertaken research on teacher emotion over the past 30 years (Figure 4). We identified 138 (89.61%) empirical studies represented the substantial majority and a subsequent 16 (10.39%) were conceptual/commentary papers. We were not surprised that no review papers were found within the Asian articles, as worldwide, only a couple of review articles have been published.

Next, an analysis of the trend of research type using the 5-year interval between 1988 and 2017 was performed. A steady rise was apparent in numbers within the two categories of articles in each interval over time with two fluctuations. It can be concluded that over the past 30 years, researchers preferred to conduct empirical studies, which therefore accounts for the major increasing trend. Although relatively small in number, over the most recent 15 years, a promising diverse trend of conceptual/commentary studies can be found. There are few review papers, however, which causes an imbalance of intellectual structure in Asia.

### Research Methods

The other aim of this study was to track research methods employed by scholars authoring 138 (89.61%) empirical studies in this Asian corpus. Empirical studies employed qualitative, quantitative, or mixed methods. The current review included a significant number of qualitative and quantitative studies. As a result, two follow-up analyses of relevant aspects for each method were constructed. We ascertained that empirical papers employed qualitative, quantitative, or mixed methods. Furthermore, for articles which employed quantitative or mixed methods, we reduced it down to five different levels of statistical methods. Third, for papers which employed qualitative and mixed methods, the different types of qualitative data collection techniques were examined.

Initially, empirical papers which employed qualitative, quantitative, or mixed methods of research were classified. Predominately, it was found that scholars who internationally studied teacher emotion, strongly preferred employing quantitative methods in their research. For the 138 empirical studies investigated in this current review, 101 (73.19%) utilized quantitative methods, 33 (23.91%) qualitative methods, but only four (2.90%) employed mixed methods.

**Figure 5:**
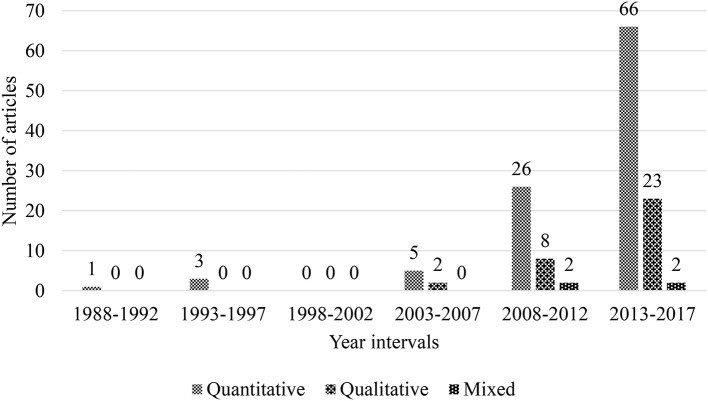
Trend of research method by the 5-year interval during 1983–2017 (*n* = 138).

Development trends using these three types of research methods were also analyzed in the 5-year interval in the past 30 years (Figure 5). A stable growth was found for studies which employed each type of research method over time. In the first four 5-year intervals, only nine (6.51%) quantitative papers were found. Subsequently, an extraordinarily sharp increase was found in the number of papers from the fourth (5, 3.62%) to fifth (26, 18.84%) interval. The number of publications in the last period almost tripled (66, 47.82%). In the fourth 5-year interval, qualitative articles appeared much later than quantitative ones, 2 (1.45%). In the sixth period, this number increased to 8 (5.80%), and in the last 5 years, finally grew to 23 (16.67%). Articles that employed a design using mixed methods started to show up in the fifth interval with two articles (1.45%) and remained the same in the final interval.

It was also of interest that the majority of studies (132, 95.66%) used data that can be considered as first-person data (e.g., teacher), six (4.35%) employed experimental research designs, and two (1.45%) used intervention studies. It is necessary to note that cross-sectional design was and remains a characteristic of quantitative studies. In our review of 105 quantitative papers, 99 (94.29%) employed a cross-sectional methodological design and six (5.71%) used the longitudinal design. Moreover, no articles adopted reciprocal or iterative method designs.

To summarize, the number of each category of studies was small in the former four 5-year intervals. In the latter two periods, vigorous growth was found, among which, the number of quantitative articles increased most swiftly, followed by qualitative and mixed papers. First-person data prevailed.

Second, we consolidated the subset of quantitative publications so that the types of statistical methods used could be fully understood. To assist this analysis, five different levels of statistical methods were employed to code the data. This coding was based on a classification scheme used previously by Hallinger (2011). The definitions of the five levels are as follows:

Level 1: Descriptive. Using numbers to represent central tendencies and/or score variability.
Level 2: Single causal factor correlational. Examining relationships or associations between two variables, one that presumably co-varies with or influences the other.
Level 3: Single causal factor–correlational with controls. This examines relationships between two variables while controlling for the influence of one or more other variables.
Level 4: Multiple factor. This involves the probing of the differential effects of multiple sources of the influence on a particular variable.
Level 5: Advanced modeling. This comprises tests capable of exploring relationships that may exist among multiple independent and dependent variables allowing for moderating and/or mediating effects to be examined.

**Figure 6:**
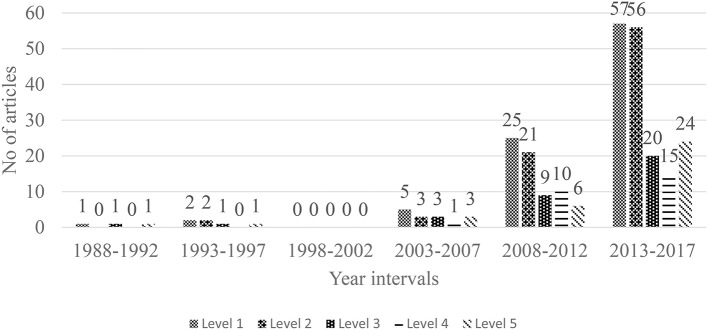
Trend of quantitative method by the 5-year interval during 1983–2017 (*n* = 105). Level 1 stands for descriptive statistical analysis method; Level 2 stands for single causal factor–correlational relationship between two variables; Level 3 stands for single causal factor–correlational with controls; Level 4 stands for multiple factor which involves probing the differential effects of multiple sources of influence on a particular variable; Level 5 stands for advanced modeling.

We made efforts to analyze the development trends of the use of five levels of quantitative statistical analysis in the past 30 years (Figure 6). There has been a stable increase in the number of each level of statistical analyses overtime. Unexpectedly, 105 (76.09%) of the 138 empirical studies utilizing either quantitative or mixed methods, Level 1 (90 out of 105, 85.71%), ranked the highest in terms of frequency of use, followed by Level 2 (82, 78.10%), Level 5 (35, 33.33%), Level 3 (34, 32.38%), and Level 4 (26, 24.76%). The number of publications in each level increased in the last ten years.

Next, an analysis of the trend of statistical level by the six 5-year intervals was conducted. Likewise, with the general trend over the past 30 years, articles using each category of statistics did not show up in large numbers until the fifth 5-year interval. For example, only one paper (0.96%) was published in the first period and five (4.81%) in the fourth period. Afterwards, the number of these articles increased in the last two periods (25, 24.04% in the fifth and 57, 54.81% in the sixth). The publications in the other levels shared similar trends to those in the first level.

Third, the types of techniques used to collect qualitative data (33, 21.43%) and mixed method studies (4, 2.60%) (Table 2) over time were examined. We then analyzed the types of techniques used to collect data by the 5-year interval. Since the first qualitative study of teacher emotion in Asia appeared in 2004, techniques employed to collect qualitative data collection in the latter three 5-year intervals were examined. In the fourth period, two studies (1.30%) used an interviewing and one study employed a focus group and qualitative survey, as data collection techniques. In the following intervals, there were four studies (2.60%) which collected data through interviews. A case study had first been applied to gather qualitative data in that period (6, 3.90%). Besides, one paper (0.65%) showed up which adopted classroom observation as the data collection method. In the last 5 years, the number of studies employing the interview techniques increased remarkably to 18 (11.69%). There were five papers (3.25%) which used a case study, two papers (1.30%) used observation, and one paper (0.65%) used a qualitative survey to collect data. In addition, four studies (2.60%) applied document analysis as the qualitative data collection technique. To summarize, over the past 15 years, interviewing has been the most widely used data collection technique in qualitative research, followed by case study, document analysis, observation, qualitative survey, and focus group.

**Table 2:** Research foci by qualitative data collection method by six 5-year intervals.

**Year interval**	**Interview**	**Case study**	**Document analysis**	**Observation**	**Qualitative survey**	**Focus group**
1988–1992						
1993–1997						
1998–2002						
2003–2007	2				1	1
2008–2012	4	6		1		
2013–2017	18	5	4	2	1	
Total	24	11	4	3	2	1

In summary, this research method section has demonstrated a thorough examination of the research types of the empirical studies, the statistical levels employed by the quantitative studies, and the data collection techniques used by qualitative research studies. The analysis shows that review studies need to be conducted and conceptual/commentary review should be paid more attention to; Level 3, 4, and 5 statistical analysis methods can be run more frequently; and additional different techniques (e.g., observation, focus group, etc.) should be applied to gather qualitative data.

### Research Topics and Types

We scanned the developmental trend of research foci by research type over the past 30 years. The majority of publications on the three research foci were empirical studies. Particularly, there were 32 articles on the first research theme, the nature of teacher emotion, among which, 22 were empirical studies and 10 conceptual/commentary review articles. Likewise, 114 papers on the relationship, the second research topic theme were all empirical studies. There were 17 articles which examined the emotional capacity of teachers, among which, 10 articles were empirical studies and seven were conceptual/commentary reviews.

The developmental trend between research foci and research type in six 5-year intervals were also examined (Table 3). Among 32 articles on the theme of the nature of teacher emotion, the first paper (3.13%) appeared in the first 5-year interval which was an empirical study. There was one empirical and one conceptual/commentary paper (6.25%) published in the first 5-year interval, one conceptual/commentary paper (3.13%) in the third period, and four conceptual/commentary papers (12.50%) in the fourth period. However, the larger proportion of the articles were published in the last two 5-year intervals, six empirical studies and one conceptual/commentary paper (21.88%) in the fifth interval and 15 empirical studies and three conceptual/commentary papers (56.25%) in the sixth period.

**Table 3:** The developmental trend between research foci and research type by six five-year intervals.

	**Nature of teacher emotion**		**Various models regarding teacher emotion**	**Teachers' emotional capacity**	
	**Empirical**	**Conceptual**	**Empirical**	**Empirical**	**Conceptual**
1988–1992	1	1			
1993–1997			3		1
1998–2002		1			
2003–2007		4	6	1	
2008–2012	6	1	26	5	3
2012–2017	15	3	79	4	3
Total	22	10	114	10	7

Comparatively, the number of articles on the second research foci, relationship, were larger than those on the first theme in the first four five-year intervals, three (2.63%) in the second period and six (5.26%) in the fourth. The number of relationship studies increased rapidly to 26 (22.81%) in the fifth interval and 79 (69.30%) in the last interval. The third research topic on emotional capacity research showed up in the second five-year interval, one conceptual/commentary paper (5.88%) published in 1997. In the fourth period, one (5.88%) empirical paper on emotional capacity appeared; then the number of this category of studies in the fifth interval increased remarkably to eight (47.06%), five empirical studies and three conceptual/commentary papers stayed stable in the last 5 years, then increased to seven (41.18%), four empirical studies, and three conceptual/commentary papers.

In summary, empirical studies were a major research type in each research theme, especially in the second theme of the relationship between teacher emotion and other constructs. This feature was also apparent in the last 5-year interval. Moreover, each type of article in each research theme did not show up in great numbers until the last two 5-year intervals, which probably benefited from the voluminous publications which appeared in this research area during the past 5 years.

### Research Topics and Methods

To unveil the patterns between research topics and research methods on teacher emotion literature, we conducted three analyses. First, we explored the patterns between research topic (i.e., Theme 1–the nature of teacher emotion, Them 2–various models regarding teacher emotion, and Them 3–teachers' emotional capacity) and research methods (i.e., qualitative, quantitative, and mixed methods). Second, we examined the research foci by five levels of statistical methods for publications with quantities and mixed methods. Third, we tracked the research foci by different kinds of qualitative data collection techniques for papers employing qualitative and mixed methods.

First, we traced the developmental trend between three research themes by research method (quantitative, qualitative, and mixed) over time. Generally speaking, data showed that the papers on the three themes with quantitative methods presented earlier and prevailed over the past 30 years compared with the other two research methods. Among the 32 articles on the nature of teacher emotion, seven papers (21.88%) were quantitative studies, 15 (46.88%) qualitative, and one (3.13%) mixed. As for the second research topic theme, the largest proportion of the relationship research included quantitative studies (88, 77.19%), followed by 23 papers (20.18%) employing qualitative research methods and three (2.63%) using the mixed methodological research design. In the pool of 17 emotional capacity studies, seven articles (41.18%) included quantitative research, and three papers (17.65%) used qualitative methods.

The patterns between research foci and research method over six 5-year intervals were investigated (Table 4). A similar trend was found over the six 5-year intervals. More specifically, there was one paper (3.13%) in the first research foci that employed quantitative method in the first period, two (6.25%) in the fifth period, and five (15.63%) in the sixth period. Qualitative research on the nature of teacher emotion did not appear until the last 10 years, five studies (15.63%) in the fifth, and 10 (31.25%) in the sixth interval. There was only one article (3.13%) which adopted a mixed method design in the fifth 5-year interval. When examining the second research theme on relationship studies employing quantitative methods, data showed three papers (2.63%) in the second period, and 21 (18.42%) in the fifth interval, and 60 (52.63%) in the sixth interval. By contrast, qualitative research on relationship issues did not appear until the last three periods, two studies (1.75%) in the fourth interval and 17 (14.91%) in the sixth interval. There was only one article (0.88%) which adopted a mixed methodological research design in the fifth 5-year interval and two (1.75%) in the last 5 years. With regard to the third research theme on emotional capacity by employing quantitative methods, one paper (5.88%) in the fourth period and two (11.76%) in the sixth interval were also spotted. Qualitative research on emotional capacity did not appear until the last two intervals, one study (5.88%) in the fifth interval, and two (11.76%) in the sixth interval.

**Table 4:** Developmental trend between research foci and research method by six five–year intervals.

	**Nature of teacher emotion**			**Various models regarding teacher emotion**			**Teachers' emotional capacity**		
	**Quantitative**	**Qualitative**	**Mixed**	**Quantitative**	**Qualitative**	**Mixed**	**Quantitative**	**Qualitative**	**Mixed**
1988–1992	1								
1993–1997				3					
1998–2002									
2003–2007				4	2		1		
2008–2012	2	5	1	21	4	1	4	1	
2013–2017	5	10		60	17	2	2	2	
Total	7	15	1	98	23	3	7	3	0

To summarize, it seems that both quantitative and qualitative research methods were more likely to be employed but mixed methods were used less by the three themes over the examined period. Quantitative methods were often used in the articles of Theme 2 (e.g., relationship) and Theme 3 (e.g., teacher emotional capacity) which was especially the case in the last two intervals. Whilst qualitative methodology was used more often in the first theme of teacher emotion nature studies, mixed methodological research design in the first two themes only appeared during the last two intervals but did not contribute to the publications in Theme 3. Hence, mixed methods need to be especially encouraged.

Second, we examined the research foci by five levels of statistical methods over the past 30 years. Generally, in each research theme, the largest proportion of the published works were employed the first level and the second levels of quantitative analysis methods. Particularly, among those eight quantitative articles on the nature of teacher emotion, seven papers (87.50%) used Level 2 statistical methods, five (62.50%) used Level 1, four (50.00%) used Level 5, two (25.00%) used Level 3, and one (12.50%) used Level 4. Among 91 studies on the second theme of relationship employing quantitative research design, 78 articles (85.71%) adopted Level 1 statistical methods, 72 (79.12%) Level 2, 31 (34.07%) Level 3, 30 (32.97%) Level 5, and 25 (27.47%) Level 4. Among those seven quantitative studies on emotional capacity, there were seven papers (100.00%) which applied Level 1 statistical analysis methods, six (85.71%) Level 2, two (28.57%) Level 3, and 2 (28.57%) Level 5.

Then, we conducted an analysis of the three research themes by quantitative research methods over six 5-year intervals (Table 5). Most of the studies in each research theme in each 5-year interval employed Level 1 and Level 2 statistical methods over the past 30 years. There was only one paper (12.50%) which used Level 3 in the first and the sixth intervals. There was one article (12.50%) which applied Level 4 in the fifth period, one study (12.50%) used Level 5 in the first and the fifth interval, and two (25.00%) in the sixth interval. Similar developmental patterns are shared by the other two research foci.

**Table 5:** Developmental trend between research foci and quantitative method by six five-year intervals.

	**Nature of teacher emotion**					**Various models regarding teacher emotion**					**Teachers' emotional capacity**				
	**Level 1**	**Level 2**	**Level 3**	**Level 4**	**Level 5**	**Level 1**	**Level 2**	**Level 3**	**Level 4**	**Level 5**	**Level 1**	**Level 2**	**Level 3**	**Level 4**	**Level 5**
1988–1992	1		1		1										
1993–1997						2	2	1		1					
1998–2002															
2003–2007						4	2	2	1	3	1	1	1		
2008–2012	1	2		1	1	20	17	8	10	4	4	4	1		2
2013–2017	3	5	1		2	52	51	20	14	22	2	1			
Total	5	7	2	1	4	78	72	31	25	30	7	6	2	0	2

In summary, each level of quantitative analysis method was used in studies on each theme, while Levels 1 and 2 were employed more frequently in each theme of research. For each level of quantitative analysis methods, articles on each theme did not appear in large numbers until the last 5-year interval.

Third, we then observed the research foci by different types of qualitative data collection techniques over the past 30 years. Generally, most of the studies in each category of research themes employed interview or case study techniques to collect qualitative data. In detail, among the 15 studies with qualitative methods on the nature of teacher emotion, 12 papers (80.00%) used interview as the data collection technique, three (20.00%) employed case study, one (6.67%) observation, and two (13.33%) document analysis. Among the 26 studies on relationship between teacher emotion and other variables, 16 articles (61.54%) applied interview techniques to collect qualitative data, seven (26.92%) used case study, four (15.38%) used documents, two (7.69%) observation and qualitative survey, respectively, and one (3.85%) used a focus group. Among the three emotional capacity studies employing qualitative data, all articles (85.71%) applied the interview technique.

We also conducted an analysis of the three research themes using the qualitative data collection method over the six 5-year intervals (Table 6). Likewise, the largest proportion of the qualitative studies in each research theme in each 5-year interval employed interview and case study techniques to collect data. Among the 15 qualitative studies on the nature of teacher emotion, five articles (33.33%) used interview to gather data starting in the fifth period and seven (46.67%) in the sixth interval. There were three papers (20.00%) which applied case study and three papers (20.00%) used document analysis to collect data in the sixth interval, respectively. There was only one study (6.67%) that adopted observation as the data collection technique in the fifth interval. As for the last two research foci, the patterns of frequency of use of interview and case study are similar to the first foci.

**Table 6:** Developmental trend between research foci and qualitative data collection method by six five-year intervals.

	**Nature of teacher emotion**				**Various models regarding teacher emotion**						**Teachers' emotional capacity**
	**Interview**	**Case study**	**Document analysis**	**Observation**	**Interview**	**Case study**	**Document analysis**	**Observation**	**Qualitative survey**	**Focus group**	**Interview**
1988–1992											
1993–1997											
1998–2002											
2003–2007					2					1	
2008–2012	5			1	2	3			1		1
2013–2017	7	3	3		12	4	4	2	1		2
Total	12	3	3	1	16	7	4	2	2	1	3

To summarize, the different kinds of qualitative research design were used in the first two themes excluding the theme of teachers' emotional capacity from the last two intervals. Accordingly, for each category of qualitative data collection methods, the articles on each research theme did not appear in large numbers until the last 10 years, which probably resulted from the fact that publications in this field increased in the past decade. While interview was the most frequently used qualitative data collection technique in articles on each type of research theme over time.

## Discussion

This descriptive quantitative analysis on teacher emotion in Asia was undertaken to generate a broader picture of the evolving knowledge base on research foci and research type and method in the region of Asia. In this section, the major results are outlined and interpreted.

### Research Foci

It was interesting to note that the “Asian knowledge base” showed a somewhat unexpected concentration on a relatively small number of topics. These included the nature of teacher emotion, the precursors of teacher emotion, and the effects of teacher emotion. Data showed that research foci in teacher emotion were dominated by two kinds of models or relationships, namely, precursor-effects (39, 25.32%) (e.g., Shami et al., 2017; Zysberg and Maskit, 2017) and direct-effects (50, 32.47%) (e.g., Asrar-ul-Haq et al., 2017; Krishnan and Kasinathan, 2017) over time. The trends of foci also reflected that scholars gradually realized the effects of teacher emotion in the last 10 years. This finding aligns with those in two recent reviews. One by Uitto et al. (2015) and the other by Fried et al. (2015). However, neither of the two reviews unveiled patterns and trends over time. In contrast, although the least prevalent research foci, the investigation of nature of teacher emotion seems to have increased in the past 5 years (e.g., Akbari et al., 2017; Chen, 2017). This review outlined the definition, elements, and measurements of teacher emotion (e.g., Chen, 2016; Alpaslan and Ulubey, 2017) which decried its scarcity, but no reviews or literature divulged relevant information (Berkovich and Eyal, 2015; Fried et al., 2015; Šarić, 2015). The second least attractive foci were found to be the mediation model and reciprocal model of teacher emotion. Stripping down to the first level of research foci, data also revealed the prevalence of research foci at the lower level. The topics regarding the effect of teachers' emotional capacity (e.g., Amirian and Behshad, 2016; Yin et al., 2017), the effect of teacher emotion regarding themselves and students (e.g., Lee and Vlack, 2017; Sakiz, 2017) seemed to be ubiquitous. On the contrary, the effect of teacher emotion on other stakeholders and the wider environment have been investigated deficiently. The influence of teachers' unmalleable and malleable factors, and contextual factors on teacher emotion are also scarce in the past 30 years.

### Research Types

A number of fascinating findings were identified with regard to types of research. For example, we found only two types of research, namely, empirical, and conceptual/commentary. During the past 30 years, researchers have predominantly used empirical studies (138, 89.61%) (e.g., Chen, 2017; Shami et al., 2017) which explain the substantial increase over the last 10 years. As noted, although being relatively small, a promising trend with regard to an increased number of conceptual/commentary studies (e.g., Miyagamwala, 2015; Tsang, 2015) were located in our review that somewhat balanced the wide range of article types. However, it also identified few expected review papers in Asia especially in the near future. It is also worthwhile noting that the majority of articles (132, 85.71%) used first person responses (teacher) (e.g., Akbari et al., 2017; Asrar-ul-Haq et al., 2017). In contrast, only a couple of studies (6, 3.90%) utilized an experimental research design (e.g., Karimzadeh et al., 2012; Madaliyeva et al., 2015) and fewer (2, 1.30%) were intervention studies (e.g., Babad, 1990; Siu et al., 2014). The Asian literature in the current study mirrored many features identified in relevant studies (e.g., Šarić, 2015; Uitto et al., 2015).

### Research Methods

Although authors demonstrated an overall preference for using quantitative research methods over time, the use of qualitative and mixed methods in research evidenced a marked increase during the last 10 years. Moreover, analysis of the subset of quantitative studies found that a larger than expected percentage of scholars employed advanced statistical methods, especially in the last 10 years. Furthermore, out of these publications, the cross-sectional design (125, 81.17%) (e.g., Asrar-ul-Haq et al., 2017) was prevalent. The types of qualitative data collection techniques were also scrutinized in the analysis. Data showed that since 2003, there have been more and more kinds of qualitative collection techniques. When the qualitative data collection techniques were examined in detail, interview (30, 83.33%) (e.g., Akbari et al., 2017) and case study (10, 27.78%) (e.g., Yuan and Lee, 2016; Koysuren and Deryakulu, 2017) were initially prevalent and dominated over time, whilst over the last 5-year interval a total of six qualitative data collection techniques appeared, with the trend appearing to continue.

These messages provide a general picture on the patterns of research methods on teacher emotion literature in Asia. The encouraging news is that the use of quantitative and qualitative techniques tends to be diverse over time and more advanced quantitative techniques have appeared in the literature in the last few years. However, these trends represent an imbalanced development regarding research methods. It is very interesting that a reverse trend has been observed in the renowned review study on school leaders' emotions by Berkovich and Eyal (2017). In particular, their study identified that about 60% of the research utilized the quantitative approach and 30% employed the quantitative approach. It was also observed that a lack of innovative qualitative techniques were used, for example, innovative meant measuring teachers' finger temperature, heart rate, blood pressure, and skin conductance level, which has also been shown by other scholars (Šarić, 2015; Uitto et al., 2015). It is also worth noting that only a small number of publications (4, 2.60%) used a mixed-method approach (e.g., Zembylas et al., 2011; Dolev and Leshem, 2016). In addition, the majority of findings of the reviewed literature with a cross-sectional design may need to be further tested since the cross-sectional design can only represent a temporary relationship. These findings further refine our picture of the Asian research context as being broadly immature but with emerging capacity and pockets of research excellence in terms of research methods.

### Research Foci, Types, and Methods

One of the innovative findings in this review is that trends of research foci by research type and method were observed. It is logical that any research foci can be found in empirical studies because of the large proportion of this type. However, the close examination for the ratio of research type for each foci theme may reveal some interesting patterns. Comparatively, empirical types of articles seem to focus on the foci themes of relationships followed by emotional capacity and the nature of teacher emotion, whilst the conceptual/commentary type of articles include more articles on the foci themes of the nature of teacher emotion and teacher emotional capacity. The ratio in the past 30 years discloses that this pattern has prevailed over time and will continue in the coming years.

Analysis of research foci and research methods revealed that research topics did demonstrate a preference in the use of research method. For example, the top two research foci were inclined to employ quantitative research method designs consisting of teacher emotional capacity and relationship studies, while research foci tended to utilize qualitative research method designs and the nature of teacher emotion, although quantitative research methods dominated as a whole. Scholars also showed their favor for different levels of quantitative research methods when investigating different research topics. The most advanced modeling statistical techniques tended to adopt the research theme of relationships between teacher emotion and other variables, whilst the research topic of emotional capacity was more likely to use lower levels of statistical techniques. It was interesting to observe that qualitative techniques including interview or case study were frequently used in the three research themes and the diversity of the use of qualitative techniques seemed to be increasingly obvious in recent years. However, clearer patterns were not apparent for research themes and qualitative techniques which may have been caused by a relatively small number of qualitative studies on teacher emotion literature.

As common sense in knowledge production, qualitative research designs appeared at the first stage and built a solid foundation for understanding and conceptual construction (Edmondson and McManus, 2007; Berkovich and Eyal, 2017). However, it may have been the case for knowledge production of teacher emotion in the current review in Asia since it has been identified that quantitative research designs were first employed. From this angle, the knowledge base in teacher emotion seems to be at its beginning stages (Edmondson and McManus, 2007). As for the other two research themes, relationships between teacher emotion and emotional capacity, quantitative techniques were strongly favored. This kind of preference, in terms of individual research themes, may be caused by traditions and/or profit and avoid the loss of scholars of particular research themes (Fried et al., 2015; Šarić, 2015). Whatever the reasons are, the diversity and balance of research type and methods in each research theme are desirable in order to contribute to the development of knowledge production on teacher emotion worldwide and in Asian contexts (Edmondson and McManus, 2007).

### Recommendations for Future Research

The major findings and trends on research foci, type and method have been outlined. Recommendations for future research are accordingly proposed. First, the lesson learned from the major findings of these trends is realizing the importance of prioritizing the research foci agenda. We acknowledge, however, that it is also desirable when choosing research topics to have a broad and balanced coverage (Hallinger and Chen, 2015) in building a regionally relevant knowledge base in Asia (Hallinger, 2018; Hallinger and Hammad, 2019). Learning and knowledge gained from past experiences supports the necessity further of programmatic research for understudied topics, namely, definition, elements, and measurements of teacher emotion, mediation model, and reciprocal model of teacher emotion, in order to balance the intellectual structure of research foci in teacher emotion. Progress in answering significant concerns needs sustained attention on such problems, and also be reprised by other researchers (e.g., Fried et al., 2015; Uitto et al., 2015).

Second, Asian literature is highly skewed by contributions from a preferably large number of empirical studies over time, a small number of conceptual/commentary studies, and no review studies further reflect the immature structure of the knowledge base of teacher emotion in Asia (Edmondson and McManus, 2007). Such features lead to the imbalance of intellectual structures with regard to research types in Asia, but also further limit our characterization of “Asian literature” in teacher emotion and frame the future challenges to stimulate both research capacity and knowledge production (Hallinger, 2018; Hallinger and Hammad, 2019). Considering the stage of evolution of international scholarship, the focused production of empirical research appears to be reasonably appropriate. It has been discussed and argued that a solid knowledge base must be constructed upon a considerable set of high-quality empirical authentications (Hallinger and Bryant, 2013). However, for mature knowledge production in teacher emotion to be gained, additional publications that have a conceptual research type should be advocated. Moreover, a pressing requirement for systematic reviews of research needs to be conducted in order to influence the advancement of a healthy intellectual structure, and also to generate “regional literature” to ensure that research, polices, and practices are improved (Fried et al., 2015).

Third, mixed-method and qualitative studies need to be promoted to increase the credibility and variation of the findings of research studies (Creswell and Clark, 2017), but also to contrite a mature growth of knowledge base in teacher emotion. Likewise, research methods also appear to need balanced diversification in terms of the use of quantitative, qualitative, and mixed methods. The evidence also reminds us that studies employing a longitudinal and reciprocal modeling design need to be encompassed in future agendas as the majority of current research methods focus on cross-sectional design. This, in turn, will mature scholars' research capacity and relevant research methods in formulating a teacher emotion knowledge base. As for qualitative design, prevalent techniques for collecting qualitative data are usually retrospective and reflexive (e.g., group discussion interview, life experience), which may not be as accurate as researchers might expect since such techniques are dependent on how accurate and vivid an individuals' memory is (Phelps and Sharot, 2008). Therefore, more innovative methods (e.g., observation, machine monitor) should be actively promoted in order to accumulate first-hand and immediate emotional experiences in authentic real-life circumstances (Šarić, 2015; Uitto et al., 2015). Intellectual progress depends on the correct selection and application of research methods to answer high impact research questions (Bridges, 1982). Although the literature described in this review appears to evidence some desirable characteristics, the relatively uneven distribution of research methods may prevent mature knowledge production in Asia. Hopefully, strategic efforts will accelerate knowledge production in Asia (Hallinger and Hammad, 2019).

Fourth, it can be ascertained that particular research types and methods prefer certain research foci. For example, empirical types of articles seem to focus on the foci themes of relationships followed by emotional capacity and the nature of teacher emotion, whilst conceptual types of articles are more likely to study the last two themes. Furthermore, teacher emotional capacity and relationship studies are inclined to employ quantitative research designs, whilst the nature of teacher emotion tends to utilize qualitative research designs. In the past 10 years, more advanced quantitative designs have been apparent. This unbalanced trend represents an immature development of knowledge production in teacher emotion. More authentic, complex research designs to enhance the validity of findings and balance the diversity of research types and methods in terms of research topics should be encouraged to build a mature teacher emotion knowledge base.

## Limitations

In this review, a number of limitations are highlighted. First, one limitation which was implicitly adhered to when analyzing data focused on “the patterns of knowledge production” as opposed to “the content of the research findings” positioned in this corpus of articles. No attempt was made by the research team to characterize any occurred learning from the findings reported in the reviewed studies (Hallinger, 2018). Instead, the objectives of this review were to describe formal patterns and trends of the knowledge base that existed. In our parallel qualitative systematic review, we critically examined through in-depth analysis, fundamental findings from the studies explored, particularly with regard to topic themes and content.

Second, only journal articles written in English were included in our searches. Therefore, “national literature” published in native languages were excluded. It is recognized by the research team that patterns and trends could be seen differently if other publications previously described were included in the analysis. It would appear, however, that such a scope is not easily accomplished. A possible strategy that could be used is for scholars to conduct their own “national review” and include publications written in their native languages. As a result, findings could be combined and expose a complete picture of teacher emotion knowledge production.

It is noted that this review only included the four search terms of common description of emotion as “emotion, mood, affect, and feeling.” This might potentially lead to the exclusion of articles that studied discrete emotions such as guilt and shame. We tried to minimize this potential risk of search terms with two recommended strategies. First, we opted to use Google Scholar to conduct searches with identical keywords for peer-refereed articles which were published in the same timeframe so that any missing articles and gray literature were captured (Polanin et al., 2017). Secondly, a “snowballed” strategy was employed for the four review articles previously mentioned by searching reference lists and “cited by” references on websites found via Google in order to locate any journal articles which may not have been located through our electronic database searches (Dietrichson et al., 2017). We acknowledge this as one limitation in the current review, even though the two strategies were utilized.

To conclude, it seems that knowledge production within the field of teacher emotion in Asia is still in its infancy (Edmondson and McManus, 2007), although a growing recognition has been apparent, especially over the last 10 years. It is expected that the uneven development of research topics, research types and methods identified in this review will appeal to scholars in the field and facilitate knowledge production in Asia as well as worldwide in a mature manner.

## Author Contributions

The author confirms being the sole contributor of this work and has approved it for publication.

### Conflict of Interest Statement

The author declares that the research was conducted in the absence of any commercial or financial relationships that could be construed as a potential conflict of interest.
